# {Ba[Au(SCN)_2_]_2_}_*n*_: a three-dimensional net comprised of monomeric and trimeric gold(I) units

**DOI:** 10.1107/S1600536810021276

**Published:** 2010-06-09

**Authors:** A. Darren Back, Tonia L. Stroud, Cynthia A. Schroll, Nathan L. Coker, Jeanette A. Krause

**Affiliations:** a Department of Physical Sciences, Morehead State University, Morehead, KY 40351, USA; b Department of Chemistry, University of Cincinnati, Cincinnati, OH 45221-0172, USA

## Abstract

The noteworthy structural feature of the title complex, poly[acetonitrile­tetra-μ_2_-thio­cyanato-barium(II)digold(I)], {[Au_2_Ba(SCN)_4_(CH_3_CN)]}_*n*_, is that the bis­(thio­cyanato)­aurate(I) anion adopts both monomeric and trimeric motifs. The trimer unit has an Au⋯Au distance of 3.1687 (3) Å. In both the monomeric and trimeric units, the Au^I^ atoms are also bonded to two S atoms. Within the trimeric unit, the Au^I^ atoms exist in differing environments; one Au atom has a T-shaped three-coordinate geometry while the other has a square-planar four-coordinate geometry. The Au^I^ atom of the monomer adopts a linear two-coordinate geometry. The extended structure can be described as a three-dimensional coordination polymer consisting of chains of Ba atoms bridged by thio­cyanate N atoms. These chains are cross-linked *via* the gold monomeric and trimeric units.

## Related literature

For further information on gold chemistry, see: Anderson *et al.* (2007 ▶); Bondi (1964 ▶); Pathaneni & Desiraju (1993 ▶); Arvapally *et al.* (2007 ▶); Beavers *et al.* (2009 ▶); Coker (2003 ▶); Coker *et al.* (2004 ▶, 2006 ▶); Katz *et al.* (2008 ▶); Puddephatt (2008 ▶); Schmidbaur & Schier (2008 ▶); Schwerdtferger *et al.* (1990 ▶); Stroud *et al.* (2009 ▶). For a description of the Cambridge Structural Database, see: Allen (2002 ▶)
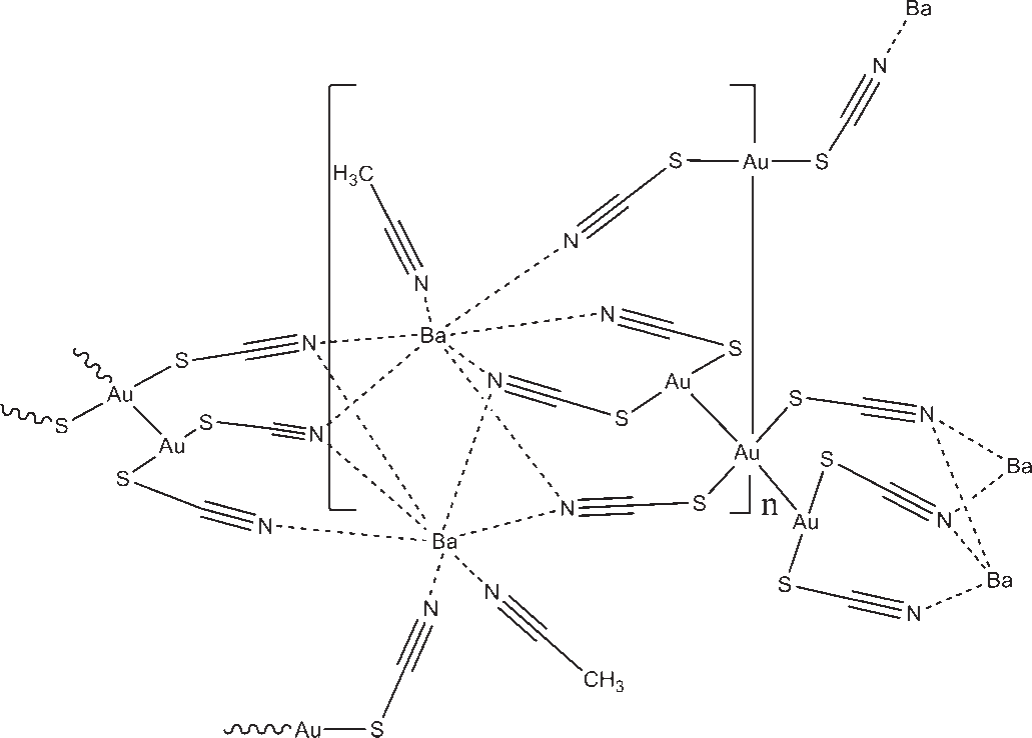


## Experimental

### 

#### Crystal data

[Au_2_Ba(NCS)_4_(C_2_H_3_N)]
*M* *_r_* = 804.65
Monoclinic, 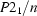

*a* = 11.5586 (3) Å
*b* = 7.4859 (2) Å
*c* = 18.5798 (4) Å
β = 106.450 (1)°
*V* = 1541.84 (7) Å^3^
*Z* = 4
Cu *K*α radiation
μ = 59.69 mm^−1^
*T* = 150 K
0.15 × 0.05 × 0.02 mm

#### Data collection

Bruker SMART6000 CCD diffractometer
Absorption correction: multi-scan (*SADABS*; Sheldrick, 2003 ▶) *T*_min_ = 0.060, *T*_max_ = 0.302
12455 measured reflections
2743 independent reflections
2559 reflections with *I* > 2σ(*I*)
*R*_int_ = 0.053

#### Refinement

*R*[*F* ^2^ > 2σ(*F* ^2^)] = 0.031
*wR*(*F* ^2^) = 0.079
*S* = 1.04
2743 reflections
167 parameters
H-atom parameters constrained
Δρ_max_ = 2.17 e Å^−3^
Δρ_min_ = −1.27 e Å^−3^

### 

Data collection: *SMART* (Bruker, 2003 ▶); cell refinement: *SAINT* (Bruker, 2003 ▶); data reduction: *SAINT*; program(s) used to solve structure: *SHELXTL* (Sheldrick, 2008 ▶); program(s) used to refine structure: *SHELXTL*; molecular graphics: *SHELXTL* and *DIAMOND* (Brandenburg, 2009 ▶); software used to prepare material for publication: *SHELXTL*.

## Supplementary Material

Crystal structure: contains datablocks I, global. DOI: 10.1107/S1600536810021276/bg2340sup1.cif

Structure factors: contains datablocks I. DOI: 10.1107/S1600536810021276/bg2340Isup2.hkl

Additional supplementary materials:  crystallographic information; 3D view; checkCIF report

## Figures and Tables

**Fig. 1.**
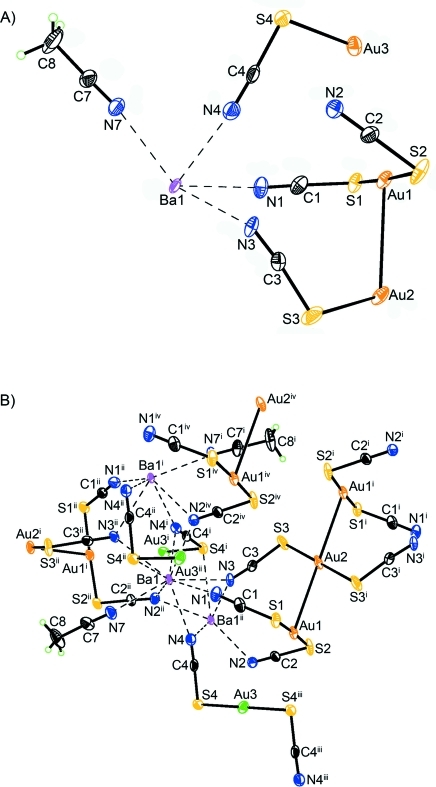
: (a) Asymmetric unit of (I). (b) Partially extended structure of (I) showing the gold monomer (Au3, drawn in green) and trimer (Au1 and Au2, drawn in orange) units and the coordination about the barium atoms. Atomic labelling scheme and 50% probability ellipsoids are shown. [Symmetry codes: (i) x-3/2, y-1/2, -z+1/2; (ii) x, y-1, z; (iii) -x+2, -y+2, -z+1; (iv) -x+1, -y+1, -z+1].

**Fig. 2.**
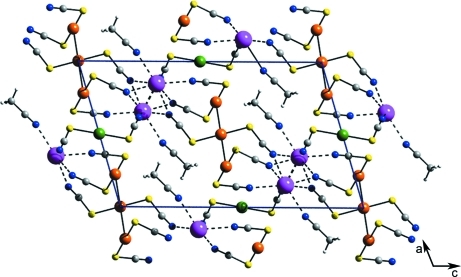
: The three-dimensional polymeric network of (I) in the ac plane (Au atoms of the monomer and trimer units drawn in green and orange, respectively.)

## supplementary crystallographic information

### Comment

The propensity for gold complexes to adopt fascinating structures, high stability and unexpected stoichiometries arising from various gold-gold interactions, termed *aurophilicty* or more generally *metallophilicity*, ultimately result in intriguing physical properties (Schmidbaur & Schier, 2008; Katz *et al.*, 2008; Puddephatt, 2008).

Over the course of our research on gold(I)-thiocyanate complexes we have observed a variety of interesting bonding motifs and luminescent properties (Coker *et al.*, 2004; Arvapally *et al.*, 2007) attributed to the [Au(SCN)_2_]^-^ anion. The [Au(SCN)_2_]^-^ anion has been observed as a monomer as well as adopting polymeric geometries. For example, in the tetraphenyl arsonium (Schwerdtferger *et al.*, 1990) and phosphonium (Coker, 2003) salts, the [Au(SCN)_2_]^-^ exists as a monomer. However, we have observed (Coker *et al.*, 2004) that the alkali (K^+^, Rb^+^ and Cs^+^) salts of this anion preferentially adopt a linear one-dimensional polymeric chain motif with Au—Au distances in the 3.0065 (5)-3.2654 (2) Å range. Ammonium or tetramethylammonium salts of bis(thiocyanato)aurate(I) exhibit a slight variation on this one-dimensional bonding theme; tending toward linear or nearly linear motifs with alternating sets of gold-gold distances; NH_4_^+^ with distances of 3.1794 (2) and 3.2654 (2) Å (Coker *et al.*, 2006) and Me_4_N^+^ with shorter distances of 3.1409 (3), 3.1723 (3) Å (Coker *et al.*, 2004). Alternatively, the [Au(SCN)_2_]^-^ anion crystallizes as a dimer unit with a gold-gold bond distance of 3.0700 (8) Å in the tetrabutylammonium salt complex (Coker *et al.*, 2004).

More recently our investigations have turned to the alkaline earth salts of gold(I)-thiocyanate. Our motivation is to further explore the influence of the cation on the highly structurally-versatile behavior of the [Au(SCN)_2_]^-^ anion. In the present work, the structure of the barium salt, (I), is presented.

The geometry of the anion in (I) (Fig. 1) is such that both monomeric and trimeric gold units are present with Au—Au and Au—S bond distances of 3.1687 (3) Å and 2.294 (2)-2.314 (2) Å, respectively. Within the trimer unit of (I), the gold atoms exist in differing environments; Au1 has a T-shaped three-coordinate geometry while Au2 has a square planar four-coordinate geometry. The monomeric gold, Au3, adopts a linear two-coordinate geometry. The S—Au1—Au2—S torsion angles, are intermediate between staggered and eclipsed (Table 1) geometries. As commented on by Pathaneni & Desiraju (1993) and further expanded by Anderson *et al.* (2007), complexes with larger Au—Au distances more frequently adopt an eclipsed conformation (L—Au—Au—L torsions ~ 0 or ±180°) presumably to lessen steric hindrance while the staggered conformation (L—Au—Au—L torsions \sim ±90°) is observed for smaller Au—Au distances. However it should be noted that there is a large spread of intermediates transitioning from eclipsed to staggered conformations with torsion angles in the ±50 to ±140° range.

The Au—Au bond distance observed in (I) is less than the sum of the van der Waals radii of 3.32 Å for a gold-gold interaction (Bondi, 1964). Furthermore, the recent literature (Schmidbaur & Schier, 2008; Katz *et al.*, 2008; Puddephatt, 2008; Anderson *et al.*, 2007) categorizes gold-gold distances in the range 2.5-3.5 Å as having significant Au—Au bonding character. In the structure presented here, the Au—Au distance is comparable to those cited for the large variety of gold complexes discussed in the literature (see, for instance, the varied works in Chem. Soc. Rev. (2008), 37, 1745–2140, a dedicated issue summarizing gold chemistry). The distance also agrees well with our work on the metallocrown complexes (dbz-18-crown-6-Ba)[bis(thiocyanato)aurate(I)] (Au—Au = 3.1109 (10) Å, Au—S = 2.288 (4)-2.305 (4) Å) (Stroud *et al.*, 2009) and [CH_3_CN-(dbz-18-crown-6-Na)]_2_[Au(SCN)_2_]_2_.dbz-18-crown-6.CH_3_CN (Au—Au = 3.0661 (4) Å, Au—S = 2.291 (2)-2.303 (2) Å) (Coker, 2003) where dbz-18-crown-6 = (6,7,9,10,17,18,20,21-octahydro- 5,8,11,16,19,22-hexaoxa-dibenzo[a,j]cyclooctadecene. The Au—S bond distances for (I) are consistent with other metal-thiocyanate complexes (average M—S distance of 2.39 Å where *M* = Pt, Pd, Ag or Au) reported in the literature (Cambridge Structural Database, Allen, 2002).

The extended structure of (I) (Fig. 2) can be described as a three-dimensional coordination polymer generated by Ba···N intermolecular interactions. As is common, a high coordination number about the barium atom in (I) is achieved. The nine-coordinate Ba atom is bound to the nitrogen atoms of the thiocyanate groups, a coordinated acetonitrile molecule (N7) and a long contact with a thiocyanate sulfur (S4) of the monomeric gold unit (Ba—N distances range: 2.777 (6)-3.0444 (7) Å; Ba···S4 = 3.422 (2) Å). The core barium bridged unit consists of a barium-barium pair (Ba···Ba = 4.4341 (5) Å) bridged by three µ-*N*(thiocyanate) groups arising from N2 and N3 of the trimer and N4 of the monomer gold units. Furthermore, these barium chains are propogated through the lattice parallel to the *b*-axis. The gold trimer units crosslink neighboring barium chains in a diagonal fashion along the [1 0 1] direction. The monomeric gold units crosslink the barium chains in a diagonal fashion along the [-2 0 2] direction completing the three-dimensional net.

A similar situation where a gold-cyanato anion, [Au(CN)_2_]^-^, adopts multiple gold bonding motifs is observed for [poly[triaquatetra-µ-cyanido-tetracyanidobis(1,4,10,13-tetraoxa- 7,16-diazacyclo-octadecane) dibarium(II)tetragold(I) (Beavers *et al.*, 2009). In that case, the coordination polymer consists of the [Au(CN)_2_]^-^ anion in monomer, dimer and trimer environments (Au—Au = 3.2670 (2)-3.5655 (2) Å) while the barium atoms are bound to the diaza-18-crown-6 and solvent water molecules (Ba···N,*O* distances span the range 2.761 (2)-2.959 (3) Å).

To echo Katz *et al.* (2008) and Puddephatt (2008), taking advantage of Au^I^*aurophilic* or other transition metal *metallophilic* interactions leads to a very useful design strategy to increase structural dimensionality of metal complexes. These complexes are highly prized due to their tunable physical properties, e.g. magnetic, vapochromic and optical properties.

### Experimental

Reaction of barium hydroxide (1 equiv) with ammonium thiocyanate (2 equiv) in water results in the formation of barium thiocyanate with the release of ammonia gas. (I) was prepared following the method described by Coker *et al.* (2004). Crystals were obtained from slow diffusion of acetonitrile-diethyl ether solution at -4 °C.

### Refinement

The H-atoms were placed in calculated positions (C—H = 0.98 Å) and treated with a riding model. The isotropic displacement parameters were defined as 1.5U_eq_ of the adjacent atom. The largest residual electron-density peaks are located near the gold trimer unit (approx. 1.6 Å from S2 and S3).

### Crystal data

**Table:**

[Au_2_Ba(NCS)_4_(C_2_H_3_N)]	*F*(000) = 1408
*M**_r_* = 804.65	*D*_x_ = 3.466 Mg m^−^^3^
Monoclinic, *P*2_1_/*n*	Cu *K*α radiation, λ = 1.54178 Å
Hall symbol: -P 2yn	Cell parameters from 7754 reflections
*a* = 11.5586 (3) Å	θ = 4.1–67.6°
*b* = 7.4859 (2) Å	µ = 59.69 mm^−^^1^
*c* = 18.5798 (4) Å	*T* = 150 K
β = 106.450 (1)°	Blade, colourless
*V* = 1541.84 (7) Å^3^	0.15 × 0.05 × 0.02 mm
*Z* = 4	

### Data collection

**Table:**

Bruker SMART6000 CCD diffractometer	2743 independent reflections
Radiation source: fine-focus sealed tube	2559 reflections with *I* > 2σ(*I*)
graphite	*R*_int_ = 0.053
Detector resolution: 0.8 pixels mm^-1^	θ_max_ = 67.6°, θ_min_ = 5.0°
ω scans	*h* = −13→13
Absorption correction: multi-scan (*SADABS*; Sheldrick, 2003)	*k* = −8→8
*T*_min_ = 0.060, *T*_max_ = 0.302	*l* = −21→22
12455 measured reflections	

### Refinement

**Table:**

Refinement on *F*^2^	Primary atom site location: structure-invariant direct methods
Least-squares matrix: full	Secondary atom site location: difference Fourier map
*R*[*F*^2^ > 2σ(*F*^2^)] = 0.031	Hydrogen site location: inferred from neighbouring sites
*wR*(*F*^2^) = 0.079	H-atom parameters constrained
*S* = 1.04	*w* = 1/[σ^2^(*F*_o_^2^) + (0.0458*P*)^2^ + 5.0574*P*] where *P* = (*F*_o_^2^ + 2*F*_c_^2^)/3
2743 reflections	(Δ/σ)_max_ = 0.001
167 parameters	Δρ_max_ = 2.17 e Å^−^^3^
0 restraints	Δρ_min_ = −1.26 e Å^−^^3^

### Special details

**Table:**

Experimental. A suitable crystal was mounted in a loop with paratone-N and immediately transferred to the goniostat bathed in a cold stream.
Geometry. All esds (except the esd in the dihedral angle between two l.s. planes) are estimated using the full covariance matrix. The cell esds are taken into account individually in the estimation of esds in distances, angles and torsion angles; correlations between esds in cell parameters are only used when they are defined by crystal symmetry. An approximate (isotropic) treatment of cell esds is used for estimating esds involving l.s. planes.
Refinement. Refinement of *F*^2^ against ALL reflections. The weighted *R*-factor wR and goodness of fit *S* are based on *F*^2^, conventional *R*-factors *R* are based on *F*, with *F* set to zero for negative *F*^2^. The threshold expression of *F*^2^ > σ(*F*^2^) is used only for calculating *R*-factors(gt) etc. and is not relevant to the choice of reflections for refinement. *R*-factors based on *F*^2^ are statistically about twice as large as those based on *F*, and *R*-factors based on ALL data will be even larger.

### Fractional atomic coordinates and isotropic or equivalent isotropic displacement parameters (Å^2^)

**Table:**

	*x*	*y*	*z*	*U*_iso_*/*U*_eq_	
Au1	0.72117 (3)	0.76278 (4)	0.517412 (17)	0.02210 (12)	
Au2	0.5000	0.5000	0.5000	0.02498 (14)	
Au3	1.0000	1.0000	0.5000	0.02274 (14)	
Ba1	0.84629 (4)	0.32246 (6)	0.29512 (2)	0.01596 (13)	
S1	0.84496 (18)	0.5362 (3)	0.57927 (10)	0.0253 (4)	
N1	0.8661 (6)	0.3726 (9)	0.4462 (4)	0.0262 (14)	
C1	0.8583 (7)	0.4404 (11)	0.4997 (4)	0.0245 (17)	
S2	0.60587 (19)	0.9988 (3)	0.45766 (12)	0.0306 (5)	
N2	0.7123 (6)	1.0410 (9)	0.3392 (3)	0.0215 (13)	
C2	0.6706 (7)	1.0228 (10)	0.3884 (4)	0.0218 (16)	
S3	0.44661 (19)	0.4332 (3)	0.37321 (11)	0.0342 (5)	
N3	0.6338 (6)	0.5268 (9)	0.3097 (3)	0.0210 (14)	
C3	0.5617 (7)	0.4914 (10)	0.3398 (4)	0.0193 (16)	
S4	1.03510 (17)	0.9889 (2)	0.38459 (11)	0.0226 (4)	
N4	0.8861 (6)	0.6993 (9)	0.3176 (4)	0.0246 (14)	
C4	0.9461 (7)	0.8143 (10)	0.3464 (4)	0.0213 (16)	
N7	1.0607 (6)	0.3524 (10)	0.2496 (4)	0.0303 (16)	
C7	1.1298 (8)	0.3672 (13)	0.2172 (4)	0.0303 (19)	
C8	1.2207 (9)	0.3905 (18)	0.1769 (5)	0.053 (3)	
H1	1.3014	0.3771	0.2120	0.079*	
H2	1.2128	0.5099	0.1544	0.079*	
H3	1.2089	0.3001	0.1373	0.079*	

### Atomic displacement parameters (Å^2^)

**Table:**

	*U*^11^	*U*^22^	*U*^33^	*U*^12^	*U*^13^	*U*^23^
Au1	0.02219 (19)	0.0297 (2)	0.01858 (19)	0.00115 (12)	0.01254 (14)	0.00146 (11)
Au2	0.0244 (3)	0.0357 (3)	0.0208 (3)	−0.00001 (18)	0.0161 (2)	0.00502 (17)
Au3	0.0219 (2)	0.0266 (3)	0.0183 (3)	0.00347 (17)	0.00345 (18)	−0.00422 (16)
Ba1	0.0183 (2)	0.0182 (2)	0.0161 (2)	0.00030 (16)	0.01252 (17)	0.00024 (15)
S1	0.0277 (10)	0.0341 (11)	0.0173 (9)	0.0060 (8)	0.0114 (8)	0.0013 (8)
N1	0.034 (4)	0.030 (4)	0.016 (3)	0.008 (3)	0.010 (3)	0.002 (3)
C1	0.024 (4)	0.030 (4)	0.022 (4)	0.007 (3)	0.009 (3)	0.009 (3)
S2	0.0326 (11)	0.0418 (12)	0.0264 (11)	0.0127 (9)	0.0229 (9)	0.0109 (8)
N2	0.026 (3)	0.023 (3)	0.018 (3)	−0.003 (3)	0.010 (3)	−0.001 (2)
C2	0.018 (4)	0.027 (4)	0.023 (4)	−0.003 (3)	0.008 (3)	−0.002 (3)
S3	0.0276 (10)	0.0560 (14)	0.0246 (10)	−0.0114 (10)	0.0165 (9)	0.0004 (9)
N3	0.023 (3)	0.029 (4)	0.015 (3)	0.006 (3)	0.011 (3)	0.002 (2)
C3	0.021 (4)	0.021 (4)	0.015 (4)	0.004 (3)	0.005 (3)	−0.001 (3)
S4	0.0199 (9)	0.0266 (10)	0.0226 (10)	−0.0036 (7)	0.0083 (7)	−0.0036 (7)
N4	0.024 (3)	0.024 (4)	0.028 (4)	0.002 (3)	0.012 (3)	0.000 (3)
C4	0.027 (4)	0.023 (4)	0.019 (4)	0.002 (3)	0.015 (3)	0.000 (3)
N7	0.025 (3)	0.045 (4)	0.024 (4)	−0.007 (3)	0.012 (3)	−0.006 (3)
C7	0.029 (4)	0.047 (5)	0.015 (4)	−0.011 (4)	0.006 (3)	0.000 (3)
C8	0.034 (5)	0.103 (9)	0.027 (5)	−0.014 (6)	0.019 (4)	−0.002 (5)

### Geometric parameters (Å, °)

**Table:**

Au1—S2	2.301 (2)	Ba1—S4^ii^	3.422 (2)
Au1—S1	2.305 (2)	S1—C1	1.689 (8)
Au1—Au2	3.1687 (3)	N1—C1	1.142 (10)
Au2—S3	2.314 (2)	S2—C2	1.671 (8)
Au2—S3^i^	2.315 (2)	N2—C2	1.156 (10)
Au3—S4	2.2940 (19)	S3—C3	1.677 (8)
Ba1—N1	2.777 (6)	N3—C3	1.158 (10)
Ba1—N7	2.845 (7)	S4—C4	1.690 (8)
Ba1—N2^ii^	2.868 (6)	N4—C4	1.140 (10)
Ba1—N4	2.869 (7)	N7—C7	1.134 (11)
Ba1—N2^iii^	2.899 (6)	C7—C8	1.463 (12)
Ba1—N3	2.971 (6)	C8—H1	0.9800
Ba1—N3^iii^	3.000 (6)	C8—H2	0.9800
Ba1—N4^iii^	3.044 (7)	C8—H3	0.9800
			
S2—Au1—S1	177.06 (8)	N1—Ba1—S4^ii^	75.45 (14)
S2—Au1—Au2	95.00 (6)	N7—Ba1—S4^ii^	73.29 (16)
S1—Au1—Au2	87.88 (5)	N2^ii^—Ba1—S4^ii^	69.32 (14)
S3—Au2—S3^i^	180.000 (1)	N4—Ba1—S4^ii^	126.36 (14)
S3—Au2—Au1^i^	77.71 (5)	N2^iii^—Ba1—S4^ii^	142.31 (13)
S3—Au2—Au1	102.29 (5)	N3—Ba1—S4^ii^	139.60 (12)
Au1^i^—Au2—Au1	180.000 (11)	N3^iii^—Ba1—S4^ii^	67.75 (13)
S4—Au3—S4^iv^	180.00 (9)	N4^iii^—Ba1—S4^ii^	115.49 (13)
N1—Ba1—N7	117.4 (2)	C1—S1—Au1	94.2 (3)
N1—Ba1—N2^ii^	72.97 (19)	C1—N1—Ba1	159.3 (6)
N7—Ba1—N2^ii^	136.8 (2)	N1—C1—S1	178.5 (8)
N1—Ba1—N4	75.90 (19)	C2—S2—Au1	97.4 (3)
N7—Ba1—N4	81.3 (2)	C2—N2—Ba1^v^	133.8 (6)
N2^ii^—Ba1—N4	139.29 (18)	C2—N2—Ba1^vi^	124.5 (6)
N1—Ba1—N2^iii^	136.58 (19)	Ba1^v^—N2—Ba1^vi^	100.50 (19)
N7—Ba1—N2^iii^	73.17 (19)	N2—C2—S2	178.0 (7)
N2^ii^—Ba1—N2^iii^	130.30 (9)	C3—S3—Au2	108.0 (3)
N4—Ba1—N2^iii^	63.85 (19)	C3—N3—Ba1	130.8 (5)
N1—Ba1—N3	70.83 (18)	C3—N3—Ba1^vi^	132.9 (5)
N7—Ba1—N3	143.0 (2)	Ba1—N3—Ba1^vi^	95.91 (17)
N2^ii^—Ba1—N3	79.92 (18)	N3—C3—S3	173.1 (7)
N4—Ba1—N3	65.37 (18)	C4—S4—Au3	99.9 (2)
N2^iii^—Ba1—N3	77.90 (17)	C4—S4—Ba1^v^	97.6 (3)
N1—Ba1—N3^iii^	139.16 (18)	Au3—S4—Ba1^v^	99.81 (6)
N7—Ba1—N3^iii^	68.39 (19)	C4—N4—Ba1	149.5 (6)
N2^ii^—Ba1—N3^iii^	77.89 (17)	C4—N4—Ba1^vi^	113.3 (6)
N4—Ba1—N3^iii^	141.35 (18)	Ba1—N4—Ba1^vi^	97.1 (2)
N2^iii^—Ba1—N3^iii^	84.24 (18)	N4—C4—S4	176.9 (7)
N3—Ba1—N3^iii^	131.05 (9)	C7—N7—Ba1	165.8 (7)
N1—Ba1—N4^iii^	122.71 (19)	N7—C7—C8	178.3 (10)
N7—Ba1—N4^iii^	119.52 (18)	C7—C8—H1	109.5
N2^ii^—Ba1—N4^iii^	62.04 (18)	C7—C8—H2	109.5
N4—Ba1—N4^iii^	118.15 (17)	H1—C8—H2	109.5
N2^iii^—Ba1—N4^iii^	68.49 (18)	C7—C8—H3	109.5
N3—Ba1—N4^iii^	68.12 (18)	H1—C8—H3	109.5
N3^iii^—Ba1—N4^iii^	62.93 (18)	H2—C8—H3	109.5

Symmetry codes: (i) −*x*+1, −*y*+1, −*z*+1; (ii) *x*, *y*−1, *z*; (iii) −*x*+3/2, *y*−1/2, −*z*+1/2; (iv) −*x*+2, −*y*+2, −*z*+1; (v) *x*, *y*+1, *z*; (vi) −*x*+3/2, *y*+1/2, −*z*+1/2.
